# An Exploratory Evaluation of Tyrosine Hydroxylase Inhibition in Planaria as a Model for Parkinsonism

**DOI:** 10.3390/ijms141223289

**Published:** 2013-11-26

**Authors:** David Prokai, Thinh Nguyen, Kurt Kamrowski, Ashwin Chandra, Tatjana Talamantes, Lewis R. Baxter, Laszlo Prokai

**Affiliations:** 1 Department of Psychiatry, College of Medicine, University of Florida, Gainesville, FL 32611, USA; E-Mails: davidprokai@gmail.com (D.P.); lbaxter@ufl.edu (L.R.B.); 2 Department of Pharmacology and Neuroscience, University of North Texas Health Science Center, Fort Worth, TX 76107, USA; E-Mails: thinh.nguyen43@mavs.uta.edu (T.N.); kurt.kamrowski@unthsc.edu (K.K.); ashwinchandra@my.unt.edu (A.C.); ttalaman@live.unthsc.edu (T.T.)

**Keywords:** planaria, parkinsonism, tyrosine hydroxylase, monoiodotyrosine, bromocriptine, antiparkinsonian agent

## Abstract

Planaria are the simplest organisms with bilateral symmetry and a central nervous system (CNS) with cephalization; therefore, they could be useful as model organisms to investigate mechanistic aspects of parkinsonism and to screen potential therapeutic agents. Taking advantage of the organism’s anti-tropism towards light, we measured a significantly reduced locomotor velocity in planaria after exposure to 3-iodo-l-tyrosine, an inhibitor of tyrosine hydroxylase that is an enzyme catalyzing the first and rate-limiting step in the biosynthesis of catecholamines. A simple semi-automatic assay using videotaped experiments and subsequent evaluation by tracking software was also implemented to increase throughput. The dopaminergic regulation of locomotor velocity was confirmed by bromocriptine, a drug whose mechanisms of action to treat Parkinson’s disease is believed to be through the stimulation of nerves that control movement.

## 1. Introduction

Despite the paradigm shift toward target-focused strategies, phenotypic screening has resulted in the majority of drug discoveries even very recently [1]. However, limitations of the phenotypic approach, in which the screening paradigm reflects key aspects of the disease process or symptom, include its restricted throughput and higher costs compared to target-based assays [1,2]. A possible solution to these shortcomings has been the pursuit of informative and effective disease models developed using live cells or simple intact organisms [3].

Planaria are amongst the simplest species that have a body plane of bilateral symmetry and axes of growth that radiate out from the head region. These traits enable cephalization, dorsal and ventral surfaces, medial and lateral regions, and an aggregate of neural cells in the head that form a bi-lobed brain [4]. The heads of free-living species possess optical, chemical and vibratory sensors that mediate tropism to food, chemical, electrical and vibration gradients, as well as response to light. Planaria also have attracted attention because they can regenerate lost tissue, including those of the nervous system, via their pluripotent stem cells that are distributed throughout their body [5]. The organism has neurotransmitter systems similar to humans [6], with its central nervous system consisting of cholinergic and dopaminergic neurons in balance [7]. As a result, planaria respond to dopaminergic agents such as dopamine (DA) agonists/antagonists and neuronal DA reuptake inhibitors (e.g., cocaine) with characteristic behaviors or changes in locomotor activity [8,9]. The latter has been assessed quantitatively (9), and a computerized image analysis-based method to track and evaluate locomotion phenotype of planarians has been reported recently [10].

Parkinson’s disease (PD) primarily affects the catecholamine region of the human brain, with degeneration of dopaminergic neurons in the substantia nigra and the locus coeruleus leading to the reduction of striatal DA levels [11]. Currently available therapies improve the symptoms of PD, but do not prevent neurodegeneration [12]. A model for parkinsonism to enable simple, rapid and inexpensive testing in a living system would be an attractive approach to screen compounds for potential neuroprotective activities.

Planaria have been used as a model organism to study dopaminergic neurodegeneration and neuroregeneration [4,5,13], as well as neurotoxicity of anti-PD pharmacotherapy [14]. The pharmacological approach to induce planarian parkinsonism has relied on dopaminergic neurotoxins such as 1-methyl-4-phenyl-1,2,3,6-tetrahydropyridine (MPTP) and 6-hydroxydopamine [15,16]. However, tyrosine hydroxylase (TH) deficiency is a hallmark of PD [17,18], yet inhibition of this first and rate-limiting enzyme of DA biosynthesis has not been considered as a model mimicking symptoms of PD in planaria. We report the implementation and preliminary validation of planarian parkinsonism through TH inhibition in an experimental setup based on the organism’s anti-tropism to light. This paradigm is intended for an expedited testing of agents that would potentially preserve dopaminergic nerve functions.

## 2. Results and Discussion

Inhibition of TH by 3-iodo-l-tyrosine (monoiodotyrosine, MIT) and the consequent blocking of dopamine production in planaria has been demonstrated [19]. Therefore, we considered MIT to induce planarian parkinsonism that would be manifested through compromised movement. In previous studies that assessed locomotor activity in planaria, velocity has been measured quantitatively placing each subject in a petri dish for observation individually in a well-lit room for 10 min [9]. Using this general experimental setup, a computerized image analysis-based method to track and evaluate locomotion phenotype of planarians as they move across a substrate in shallow water has been implemented recently [10]. However, throughput of experiments would be limited upon using models in which one animal is observed during a relatively long observation period. We reasoned that, taking advantage of the well-known anti-tropism of planarians to light, the velocity of movement away from a light source could be used as a novel behavioral test allowing for expedited assays performed simultaneously on multiple experimental subjects.

To determine the speed of movement away from light, we tested a simple setup in which a single planarian was placed in a “lane” of a test apparatus (e.g., utilizing the grooves in the bottom of a 20 × 25 × 5 cm plastic photo-developing tray). In these initial proof-of-concept experiments, a “standard” light gradient is created by placing a light source (e.g., a 30-cm fluorescent strip that delivers illuminance level of 300–600 lux and commonly used as the primary light fixture for an aquarium with a glass cover) above the zone in which the planaria was introduced. They then move away from the light towards the direction of less intense light. Upon introducing a test subject to one of the lanes, the time to travel a well-defined distance (5 cm) was recorded, with paying attention to possible changes in movement style, for calculation of average velocities. Measurements were performed in a dark room every 24 h for 4 days with treatment achieved by keeping, except during the maximum 2-min test periods, the subjects in a small petri dish filled with the given concentration of TH inhibitor dissolved in a standard test medium (STM, see Experimental Section). The results are summarized in Table 1.

A two-way repeated measures analysis of variance (ANOVA) considering the three MIT concentrations plus STM control and four scheduled times of evaluation indicated that the effect of treatment was statistically significant with *F*_(3,9)_ = 30.2, *p* < 0.001. On the other hand, the main effect of time yielded *F*_(3,9)_ = 0.66, *p* = 0.581, showing that the duration of MIT exposure had no significant effect on the locomotion velocity. Also of note is that the interaction effect (treatment × time) was not significant: *F*_(9,159)_ = 0.78, *p* = 0.639. No change in movement phenotype was observable even upon prolonged treatment with high concentration of the TH inhibitor (1 mM). Because only the main effect of MIT concentration was found to reach statistical significance, we selected 24-h treatment to allow for expedited experiments upon testing neuroprotective agents in this planarian model of parkinsonism.

**Figure 1.**
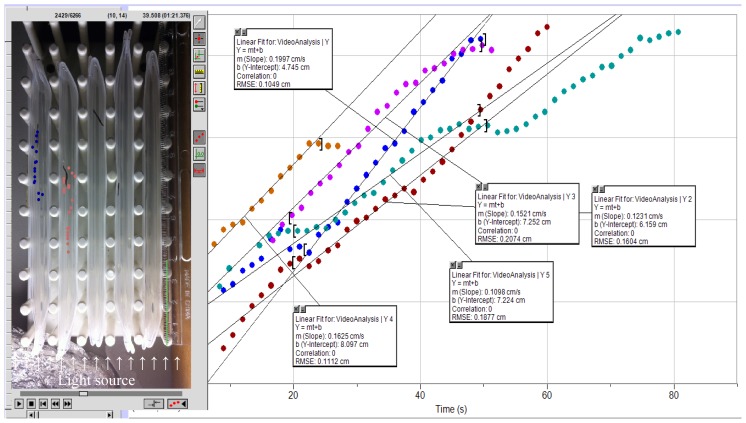
Test apparatus and principle of average velocity determination from videotaped experiments by using tracking software.

To increase throughput and facilitate multiplexing of the measurements, we folded 16 cm × 4.5 cm rectangles from 0.75-mm thick low-density polyethylene Clear Plast-O-Mat^®^ Ribbed Shelf Liner™ into boat shapes to seal them by clipping on both ends. These “boats” fit conveniently into a Nalgene^®^ peg-style rack for 14–17 mm test tubes to form, when filled with about 5 mL STM, multiple testing lanes. This simple apparatus allowed for videotaping of parallel experiments and subsequent computerized processing by commercial tracking software. The setup and examples of video analyses are shown in Figure 1. Briefly, evaluation started 20 s after the planaria’s placement into the lane and movement were tracked for 30 s at 0.67 Hz frequency followed by determination of average velocities by linear regression.

**Figure 2.**
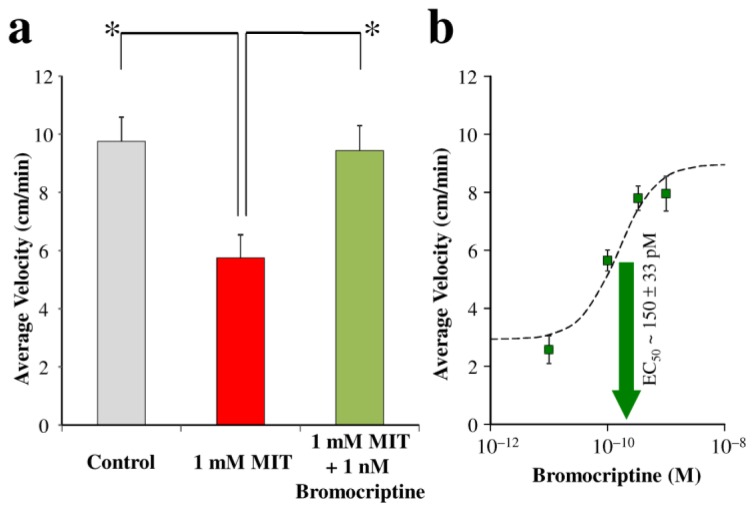
(**a**) Average velocity of planaria moving away from light without TH inhibition (grey bar, control), after 24-h treatment with 1 mM MIT (red bar), and after 24-h co-treatment with 1 mM MIT and 1 nM bromocriptine (green bar). Data are means ± standard errors, *N*/group = 5. One-Way ANOVA: *F*_(2,12)_ = 7.24, *p* < 0.009; asterisks indicate statistically significant difference by *post hoc* tests; (**b**) Treatment concentration–response curve and EC_50_ of bromocriptine in MIT-induced parkinsonism. Data are means ± standard errors, *N*/group = 8–10.

For the discovery of agents that ameliorate planarian parkinsonism, we hypothesized that they will reverse movement deficits upon co-treatment with the TH inhibitor. Bromocriptine [(5′α)-2-bromo-12′-hydroxy-5′-(2-methylpropyl)-3′,6′,18-trioxo-2′-(propan-2-yl)ergotaman] was our drug of choice to prove this hypothesis and validate our model for the screening of potential antiparkinsonian compounds. As a DA agonist, this drug is believed to provide symptomatic treatment of PD in humans not only by stimulating nerves that control movement, but also as an agent with multiple modes of action including neuroprotection elicited through various mechanisms such as l-3,4-dihydroxyphenylalanine (l-dopa) sparing, antioxidant, autoreceptor and antiapoptotic effects, as well as amelioration of subthalamic nucleus-mediated excitotoxicity [12,20]. Figure 2a shows that co-treatment with 1 nM bromocriptine was, indeed, protective against MIT-induced parkinsonism in planaria. In a separate series of experiments using this invertebrate model, we found clear association between the bromocriptine concentration used and the reversal of MIT’s inhibitory effect (Figure 2b). Presuming a sigmoidal curve for the concentration–effect relationship [21], non-linear fitting resulted in 150 ± 33 pM and 1.34 ± 0.40 as the effective concentration yielding 50% of the maximum response (EC_50_) and the Hill coefficient, respectively.

Bromocriptine is also known to elicit dyskinesia, a known side-effect of DA agonists used in the treatment of PD, in humans [20]. In planaria exposed to high concentrations (≥10 nM) of this drug alone, their locomotor velocity in our tests decreased below 1 cm/min. Moreover, they displayed both “screw-like” and “C-like” hyperkinesia. Previous studies have reported that planaria show specific behavioral patterns in response to drugs acting on neural transmission. In particular, stimulation of dopamine D1- and D2-receptors produces screw-like hyperkinesia and C-like position, respectively [8,22]. These hyperkinetic responses, which we confirmed for bromocriptine in our studies above, may represent the behavioral equivalent of stereotyped activities in mammals thereby giving additional indication about the potential value of planarian movement-based phenotypic screens in exploratory drug research.

## 3. Experimental Section

All materials were purchased from commercial vendors. Planarians (*Dugesia dorotocephala*) were obtained from Carolina Biological (Burlington, NC, USA). They were maintained in spring water, with water replenished every other day and feeding once a week. MIT was dissolved in STM (0.6 g CaCl_2_, 0.24 g MgSO_4_, 0.12 g NaHCO_3_, 0.012 g KCl in 2.0 L of distilled water). Bromocriptine stock solution (1 mg/mL) was made in dimethyl sulfoxide (DMSO) for serial dilution into STM to reach testing concentration. DMSO contents were kept well below 0.1% (*v*/*v*) in all treatment solutions, which avoided interference from the solvent in the experiments [23]. The same serial dilution from pure DMSO was used to prepare control STM and MIT solutions for comparison with the bromocriptine experiments. Exposure to experimental agents was achieved by keeping the planaria in small petri dishes that contained the agent dissolved in STM. Tests were scheduled at the same time during the day each consecutive time.

Videos were recorded using an iPhone 4S (Apple, Cupertino, CA, USA), which was held in place above the experimental apparatus (Figure 1) by using a 3-finger clamp mounted on portable laboratory stand. The recordings were transferred to a personal computer for analyses by Logger Pro (version 3, Vernier Software & Technology, Beaverton, OR, USA). Tracking of the subjects at 0.67 Hz frequency was set to start after 20 s delay and lasted for 30 s. Average velocities were estimated by linear regression.

Statistical evaluations were done by ANOVA with *p* < 0.05 considered statistically significant. Follow-up, two-group comparisons employed Student-Newman-Keuls *post hoc* tests (*p* < 0.05). EC_50_ was calculated using the Scientist software (version 2.01, Micromath, St. Louis, MI, USA) by fitting the results of the treatment concentration–response experiments to an equation similar to the one introduced by Cheng and Prusoff [21]:

(1) $$\Delta =\Delta_{\text{max}}/[1+{\left({\text{EC}}_{50}/C_{i}\right)}^{\text{h}}]$$

where Δ is the measured average increase in velocity compared to control upon exposure to bromocriptine solution of *C_i_* concentration in the STM solution, Δ_max_ is the difference of average velocities between the untreated control subjects and those treated with MIT only (1 mM), and h is the Hill coefficient.

## 4. Conclusions

We have introduced a planarian model of parkinsonism in which pharmacological inhibition of TH, the first and rate-limiting enzyme of DA biosynthesis, by MIT is used. The assay takes advantage of anti-tropism to light in this invertebrate species making measurements easy to perform relatively quickly and in multiplexed format with semi-automated evaluation. A potential application of the model as an *in vivo* screening method for antiparkinsonian agents to protect against dopaminergic functional decline has been demonstrated using bromocriptine. Further studies probing the value of our planarian model of parkinsonism are in progress.

## Figures and Tables

**Table 1.** Velocity of light gradient-induced locomotion in planaria exposed to various concentrations of monoiodotyrosine (MIT) that inhibits tyrosine hydroxylase (TH), the first and rate-limiting enzyme of dopamine (DA) biosynthesis a.

MIT Concentration (mM)	Baseline b	24-h exposure	48-h exposure	72-h exposure	96-h exposure
0 b	12.4 ± 0.3	12.7 ± 0.4	11.9 ± 0.7	13.3 ± 0.6	11.8 ± 0.4
0.001	11.8 ± 0.1	11.1 ± 0.4	11.3 ± 1.2	11.5 ± 0.6	10.6 ± 0.4
0.1	12.2 ± 0.5	7.8 ± 0.6	10.3 ± 1.7	9.4 ± 1.7	8.4 ± 0.3
1	12.9 ± 1.1	4.8 ± 0.6	7.7 ± 2.9	7.3 ± 2.3	5.9 ± 1.8

a Velocities are given in cm/min, mean ± standard error (*N* = 10);

b STM only.
